# Sperm mRNA Transcripts Are Indicators of Sub-Chronic Low Dose Testicular Injury in the Fischer 344 Rat

**DOI:** 10.1371/journal.pone.0044280

**Published:** 2012-08-31

**Authors:** Sara E. Pacheco, Linnea M. Anderson, Moses A. Sandrof, Marguerite M. Vantangoli, Susan J. Hall, Kim Boekelheide

**Affiliations:** Department of Pathology and Laboratory Medicine, Brown University, Providence, Rhode Island, United States of America; The Roslin Institute, University of Edinburgh, United Kingdom

## Abstract

Current human reproductive risk assessment methods rely on semen and serum hormone analyses, which are not easily comparable to the histopathological endpoints and mating studies used in animal testing. Because of these limitations, there is a need to develop universal evaluations that reliably reflect male reproductive function. We hypothesized that toxicant-induced testicular injury can be detected in sperm using mRNA transcripts as indicators of insult. To test this, we exposed adult male Fischer 344 rats to low doses of model testicular toxicants and classically characterized the testicular injury while simultaneously evaluating sperm mRNA transcripts from the same animals. Overall, this study aimed to: 1) identify sperm transcripts altered after exposure to the model testicular toxicant, 2,5-hexanedione (HD) using microarrays; 2) expand on the HD-induced transcript changes in a comprehensive time course experiment using qRT-PCR arrays; and 3) test these injury indicators after exposure to another model testicular toxicant, carbendazim (CBZ). Microarray analysis of HD-treated adult Fischer 344 rats identified 128 altered sperm mRNA transcripts when compared to control using linear models of microarray analysis (q<0.05). All transcript alterations disappeared after 3 months of post-exposure recovery. In the time course experiment, time-dependent alterations were observed for 12 candidate transcripts selected from the microarray data based upon fold change and biological relevance, and 8 of these transcripts remained significantly altered after the 3-month recovery period (p<0.05). In the last experiment, 8 candidate transcripts changed after exposure to CBZ (p<0.05). The two testicular toxicants produced distinct molecular signatures with only 4 overlapping transcripts between them, each occurring in opposite directions. Overall, these results suggest that sperm mRNA transcripts are indicators of low dose toxicant-induced testicular injury in the rat.

## Introduction

Toxicogenomics is the convergence of emerging technologies with conventional toxicological assays to identify molecular signatures resulting from toxic insult [1], [2]. The strength of these signatures is increased when they are linked to a phenotypic endpoint and dose-response and time course studies can further identify cause and effect relationships between changes in molecular profiles after toxicant exposure. For example, microarrays can measure gene transcript levels of the entire genome simultaneously and provide the foundation for understanding, characterizing, and predicting target-organ toxicity [3].

The testis is susceptible to a variety of therapeutic agents and environmental toxicants. Injury may be subtle and histopathological changes are undetectable at early time points, while serum hormones and semen analyses are not able to detect early changes in both pre-clinical studies and clinical trials [3]. Although serum inhibin B has been evaluated as a biomarker of testicular injury, this may not be a sensitive endpoint in rodents [3]. With this in mind, several studies have used toxicogenomic approaches to screen compounds for testicular toxicity [3]. Of note, one study utilized microarray analysis of the testis after acute exposures to four model testicular toxicants. The results suggested that even though there were no histopathological changes to the testis after the exposure, the gene expression changes were robust and reproducible, with some genes differentially expressed in all treatment groups [3]. It has yet to be determined whether these transcript changes were adverse or adaptive in nature; however, these data are important, because they underscore that transcriptomic profiling can identify different toxicant responses in the testis.

The cellular heterogeneity of the testis, in addition to the spatial-temporal intricacy of spermatogenesis, makes it a very complex tissue to study. Furthermore, assessing gene expression in the testis is an unrealistic endpoint when comparing pre-clinical animal studies and clinical trials, because testicular biopsy is too invasive. On the other hand, sperm, a pure population of cells produced by the testis, reflect spermatogenic function [4]. It is understood that the quantity and types of sperm mRNA transcripts may indicate the quality and productivity of spermatogenesis [5], potentially making them valuable indicators of testicular injury or dysfunction.

Previous studies have characterized the dose-response of 2,5-hexanedione (HD) and carbendazim (CBZ) exposure on the rat testis [6]–[9], making them model toxicants with predictable male reproductive effects. These toxicants can induce alterations in microtubule assembly and disrupt germ cell development (as reviewed by [10]). HD is the active metabolite of the common industrial solvent, *n*-hexane. Although the most significant exposures occur in occupational settings, humans are ubiquitously exposed to low levels of *n*-hexane as a chemical component of gasoline [11]. HD targets the Sertoli cell, the supportive cell within seminiferous tubules, and promotes rapid microtubule assembly by inducing tubulin cross-linking, altering microtubule-dependent transport in these cells [11]. This disturbs the germ cell niche by impeding seminiferous tubule fluid secretion. Disrupting the Sertoli cell microenvironment leads to germ cell apoptosis and ultimately testicular atrophy [9], [11]–[13]. Carbendazim (CBZ) is the active metabolite of benomyl, a benzimidazole fungicide used to prevent and eliminate fungal plant diseases [14] . Mammals are exposed to CBZ orally and it is readily absorbed and metabolized rapidly. Overall, CBZ has low acute toxicity but has many negative effects on the male reproductive system [9], [14]–[18]. CBZ is a Sertoli cell toxicant that binds to the beta-tubulin subunit of the tubulin heterodimer and inhibits microtubule polymerization [19]–[22]. This ultimately decreases the rate and stability of microtubule assembly. Sub-chronic exposure of adult male Wistar rats to CBZ resulted in many histopathological changes of the testis including atrophic seminiferous tubules, decreased germ cells, and increased sloughing in a dose dependent manner [15].

Taking advantage of the wealth of data on the specific dose-response and mechanistic action of these model toxicants, we hypothesized that applying toxicogenomic techniques to pure sperm populations would be a useful approach for identifying indicators of testicular injury. The goals of this project were to further develop a sub-chronic, low dose exposure paradigm for the model toxicant HD, to utilize toxicogenomic approaches to identify alterations to the caudal epididymal sperm mRNA transcriptome, and to test a panel of sperm transcripts as sensitive indicators of testicular injury, using another toxicant, CBZ.

## Materials and Methods

### Ethics Statement

This study was carried out in strict accordance with the recommendations in the Guide for the Care and Use of Laboratory Animals of the National Institutes of Health. The Brown University Institutional Animal Care and Use Committee (Permit Number: 0906060) approved all experimental animal protocols in compliance with National Institute of Health guidelines. Animals were euthanized using carbon dioxide asphyxiation, and all efforts were made to minimize suffering.

### Animals

Adult male Fischer 344 rats (Charles River Laboratories, Wilmington, MA) weighing 175–225 grams (approximately 56–62 days of age) were allowed to acclimate for one week prior to beginning the experiment. Throughout the study the animals were maintained in a temperature and humidity controlled AAALAC accredited vivarium with a 12 hour alternating light-dark cycle. All rats were housed in community cages with free access to water and Purina Rodent Chow 5001 (Farmer’s Exchange, Framingham, MA).

### Chemicals

HD (CAS# 110-13-4, ≥99% purity), CBZ (CAS# 10605-21-7, ≥97 % purity), and all other chemicals were purchased from Sigma Aldrich (St. Louis, MO) unless otherwise noted.

### Dose Selection

Lowest-observable-adverse-effect-level doses of HD and CBZ were chosen to produce minimal but detectable testicular injury. Based on previous studies, a 3-month exposure to 0.33% HD in the drinking water or 50 mg/kg/d CBZ delivered in a corn oil vehicle by gavage was expected to produce some minimal evidence of injury in the rat testis [6]–[9].

### Administered Dose Estimation

To estimate the dose of HD administered during both the preliminary experiment and the time course, we first calculated the average daily water intake per rat for each study and adjusted it for the amount of HD in the water (0.33% v/v). The resulting value was divided by the average starting and ending weights of the rats to yield an estimated average daily intake adjusted for the density of HD (0.973 g/mL).

### Experimental Design

**Figure 1:**
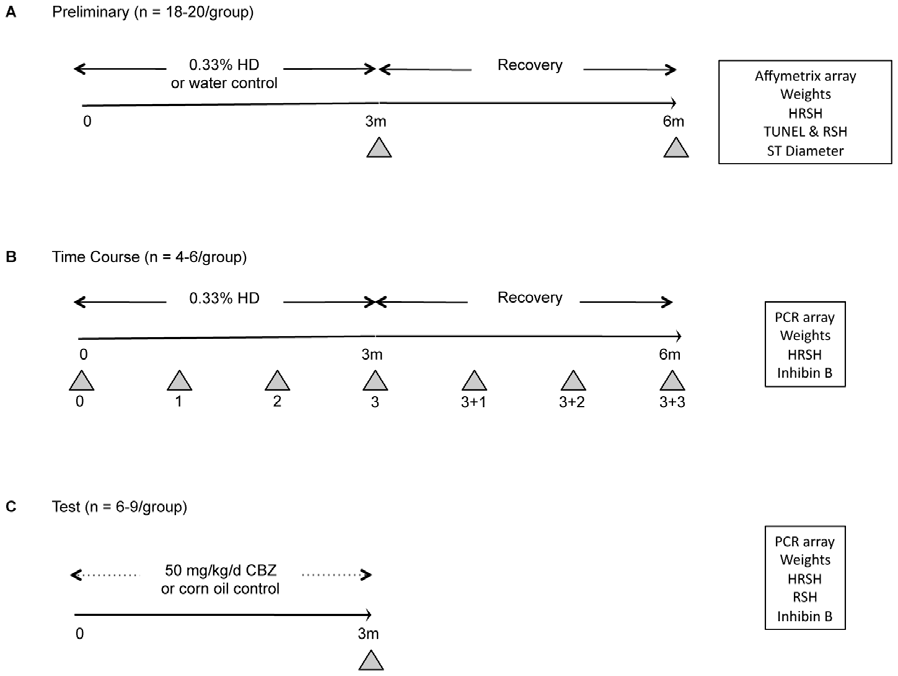
Experimental paradigms. For the Preliminary Experiment (A), rats were treated with 0.33% HD in the drinking water or water control, for 3 months. The recovery group received control water for 3 additional months. For the Time Course Experiment (B), the same study design was used as in (A), except with the addition of monthly assessments during HD exposure and post-exposure recovery. For the Test Experiment (C), rats were exposed to 50 mg/kg/d CBZ in corn oil vehicle via oral gavage (dotted lines) for 3 months. The assays conducted for each experiment are listed in the boxes on the right: HRSH = homogenization resistant spermatid head counts; RSH = enumeration of retained spermatid heads; and ST = seminiferous tubule. Grey arrowheads indicate euthanization time points.

Three experiments examined the utility of sperm mRNAs as biomarkers of toxicant-induced testicular injury: 1) a preliminary experiment using whole-genome profiling to identify a list of genes to compare control and HD-exposed sperm; 2) a more comprehensive time course experiment examining the temporal effects of HD on candidate sperm mRNA indicators; and 3) a test experiment extending this approach to evaluate CBZ, another testicular toxicant (Figure 1).

In each of the three experiments, rats were exposed to a testicular toxicant for 3 months. Rat spermatogenesis takes approximately 8 weeks, so the 3-month exposure-window guaranteed that the sperm being evaluated would have experienced a disrupted environment throughout their development, including the time needed for epididymal transport. Two of the experimental paradigms allowed rats to recover following exposure for up to 3 additional months.

Rats were euthanized by carbon dioxide asphyxiation and the body weights and reproductive organ weights were recorded at necropsy. Left testes were fixed in 10% neutral-buffered formalin for histological examination, and a portion of each animal’s right testis was detunicated and snap frozen for the automated determination of homogenization resistant spermatid head (HRSH) counts [23]. The epididymides were weighed and the caudal regions of the epididymides were used immediately for sperm isolation and RNA extraction. The following experiments were conducted:

#### Preliminary experiment

Rats were randomly assigned to four groups: water (n = 20), HD (n = 19), water-recovery (n = 18), and HD-recovery (n = 20). HD was administered as a 0.33% solution in drinking water *ad libitum* for 3 months (HD and HD-recovery groups). Rats were necropsied after 3 months of exposure (water and HD) or after 3 months of exposure plus 3 months of additional post-exposure recovery (water-recovery and HD-recovery; Figure 1A). In addition to the common endpoints assessed in all exposure paradigms (weights and testis HRSH), testis histology (including germ cell apoptosis, seminiferous tubule diameter, and spermatid head retention) was evaluated in these animals and the sperm transcriptome was characterized by microarray analysis. Exposure to 0.33% HD is known to decrease testis weight and increase stage-specific spermatid head retention in the seminiferous epithelium, so these two endpoints were selected as positive controls to confirm of testicular toxicity [8], [23].

#### Time course experiment

Rats were randomly assigned to seven groups classified by the duration of the exposure and recovery [Months (+ Recovery); 0 (n = 4), 1 (n = 6), 2 (n = 6), 3 (n = 6), 3+1 (n = 6), 3+2 (n = 5), 3+3 (n = 5)]. The same exposure paradigm was used here as in the preliminary experiment except the rats were euthanized at 1-month intervals (Figure 1B). Serum was collected from each animal at necropsy and archived for inhibin B measurement. The sperm transcript changes were confirmed using qRT-PCR array analysis.

#### Test experiment

Rats were randomly assigned to two groups, corn oil vehicle (n = 9) and CBZ (n = 6). CBZ-exposed rats received 50 mg/kg/d CBZ suspended in corn oil via oral gavage for 3 months (Figure 1C). Testis spermatid head retention and serum inhibin B levels were measured in these animals and the sperm transcript changes were determined using qRT-PCR array analysis.

### Histological Examination

Two cross sections from the center of the formalin-fixed testes were embedded in glycol methacrylate (Technovit 7100; Heraeus Kulzer GmBH, Wehrheim, Germany) for histological examination of stage-specific retained spermatid heads (RSH) (Preliminary and Application Studies) or embedded in paraffin for detection of apoptosis by TUNEL staining with concurrent measurement of seminiferous tubule diameter (Preliminary Experiment only). The Aperio ScanScope (Aperio Technologies, Vista, CA) was used to create digital images of the microscope slides and all histological endpoints were analyzed using ImageScope software.

For the enumeration of RSH, 2 sections (3 µm) of testes from 6 randomly selected rats per treatment group were stained with periodic acid-Schiff’s reagent followed by hematoxylin counter stain (PASH). Each cross section was evaluated for seminiferous tubules in spermatogenesis stages IX-XI, each of which was required to have a major:minor axis of less than 1.5∶1 [8]. The number of RSH in the basal compartment was recorded for each stage-specific seminiferous tubule, and the counts were averaged together on an individual rat basis. The counts were log-transformed to assure normally distributed errors prior to statistical analysis.

For the evaluation of apoptosis, 2 paraffin sections (5 µm) of testes from the subset of rats utilized above were stained using the ApopTag Peroxidase *In Situ* Apoptosis Detection Kit (Chemicon, Temecula, CA) following the manufacturer’s protocol and were counterstained with methyl green. Apoptotic cells were counted in 50 random seminiferous tubules having a major:minor axis of less than 1.5∶1. The percentage of seminiferous tubules containing TUNEL positive cells was assessed. The minor axis diameter was also recorded.

### Serum Inhibin B Measurements (Time Course and Test Experiments)

Blood was collected at necropsy via cardiac puncture, samples were allowed to clot before centrifugation, and serum was isolated following standard protocols. Serum samples were stored at −80°C prior to shipment to Pfizer (Groton, CT). Serum inhibin B (pg/mL) was measured using the ACTIVE® Inhibin Gen II ELISA assay kit (REF A81301, Diagnostic Systems Laboratories, Inc., via Beckman Coulter, Inc., Brea, CA) following the manufacturer’s instructions. Duplicate samples were averaged.

### Sperm Isolation and RNA Extraction

The cauda epididymides were punctured repeatedly with 30 and 26 gauge needles, placed into micro-centrifuge tubes containing phosphate buffered saline (1X PBS, GIBCO REF 10010-023, Life Technologies, Grand Island, NY), and incubated in a water bath at 37°C for 10 minutes to allow sperm release. Following centrifugation for 3 minutes at 300× g to pellet the epididymal pieces, the supernatant was removed and centrifuged for 5 minutes at 2000× g to pellet the sperm. Pellets were incubated in a somatic cell lysis buffer (0.15 M ammonium chloride, 10 mM potassium bicarbonate, 0.1 mM EDTA (Thermo Fischer Scientific Inc., Pittsburgh, PA)) for 30 seconds prior to centrifugation at 16,100× g for 1 minute to remove any somatic cell contaminants, leaving the sperm intact. The pellet was washed with PBS and centrifuged again at 16,100× g for 1 minute. RNA was extracted from the fresh sperm using the *mir*Vana miRNA Isolation Kit (Applied Biosystems/Ambion, Austin, TX). Sperm purity was confirmed by the absence of somatic cell contaminants using bright phase microscopy and by the absence of 18/28S ribosomal RNA peaks by RNA gel electrophoresis [5], [24], [25].

### RNA Cleanup and Concentration Protocols

mRNAs were further purified and concentrated into a smaller volume. In the Preliminary Experiment, mRNAs were cleaned and concentrated using ammonium acetate and ethanol precipitation. RNAs were re-suspended in 10 µl RNAse-free water. In the Time Course and Test Experiments, mRNAs were DNase treated, and processed using Qiagen’s RNase-free DNase and RNeasy MinElute Cleanup kits (Qiagen Sciences, Germantown, MD) following the manufacturer’s protocol.

### mRNA Analyses

Sperm mRNA content was assessed for each of the three experiments using microarrays or qRT-PCR arrays. The microarray data (GEO accession number GSE34641) is MIAME compliant and has been deposited in NCBI’s Gene Expression Omnibus (GEO) database [26] as detailed in the MGED Society website http://www.mged.org/Workgroups/MIAME/miame.html.

#### Preliminary experiment

Using the Brown Genomics Core Facility, the isolated sperm mRNA (160 ng/sample) from each rat (n = 18–20/group) was processed and hybridized to Affymetrix GeneChip Rat Gene 1.0 ST Arrays (Affymetrix, Santa Clara, CA). The probe cell intensity data from the Affymetrix GeneChips was normalized, annotated, and analyzed following previously published methods [25]. The microarray data were also analyzed using Ingenuity Pathways Analysis (IPA) (Ingenuity® Systems, www.ingenuity.com). The IPA Functional Analysis program identified the molecular and cellular functions that were significantly associated with the sperm mRNA transcripts. Only transcripts meeting the q-value cutoff of < 0.05 and absolute fold change values>1.5 that were recognized in Ingenuity’s Knowledge Base were considered for the analysis. For development of the qRT-PCR array (see below), twenty-nine differentially present mRNA transcripts were selected from the microarray data based on the strength of association, magnitude of change, and biological function (Table S1).

#### Time course and test experiments

Sperm mRNAs from the Time Course and Test Experiments (4–9 rats/group) were used on a SABiosciences Rat RT^2^ Profiler Custom qRT-PCR array (SABiosciences, a Qiagen Company, Frederick, MD), which profiled the expression of 32 genes× 12 samples per 384 well plate. The 32 genes included 29 candidate genes selected above plus three plate controls (GAPDH, NM_017008; RGDC, U26919; RTC, SA_00104). The 384 well PCR plates were loaded using an epMotion 5075 LH robot (Eppendorf North America, Hauppauge, NY). The qRT-PCR reactions followed the manufacturer’s instructions using an ABI 7900 HT thermocycler (Applied Biosciences, Life Technologies Corporation, Carlsbad, CA). Raw CT values were normalized to the average of the GAPDH and RGDC control CT values and expression was analyzed using the ΔΔCT method [27] following ABI’s guidelines. The fold change ratio range was generated using the formula 2^−ΔΔCT ± SE^, with the standard error generated after calculating the average ΔCT values for each transcript.

The fold changes determined in the qRT-PCR analysis for the 12 transcripts altered in the Time Course Experiment were uploaded into Multi-experiment Viewer (MeV version 10.2) [28]. Gene tree hierarchical clustering was performed using Manhattan Distance and Average Linkage Clustering.

### Statistical Analyses

#### Preliminary experiment

For body and organ weights and histological endpoints, Student’s unpaired two-tailed t-tests were performed using the Prism 5 software (GraphPad Software, La Jolla, CA) to determine differences between treated and control (p<0.05). The linear models for microarray analysis (LIMMA) procedure [29] (R package limma) was used to fit a linear regression model for each transcript on the Affymetrix GeneChip Array for each treatment and its control. The p-values were adjusted for multiple comparisons with the qvalue package in R [30], and the log_2_ expression ratios were transformed into fold change values. Transcripts were considered statistically significant when q-value < 0.05. IPA performed a right-tailed Fisher’s exact test to calculate a p-value determining the probability that each biological function assigned to the sperm transcripts was due to chance. These p-values were adjusted for multiple comparisons within the IPA software using the Benjamini-Hochberg correction. The high-level functional categories with adjusted p-values < 0.05 were considered significant.

#### Time course experiment

One way-ANOVA with Dunnett’s correction for multiple comparisons was performed using the Prism 5 software for the weights, the histological endpoints, and the mRNA qRT-PCR array data. Data was considered significant when p<0.05.

#### Test experiment

Student’s unpaired two-tailed t-tests were performed using the Prism 5 software to determine statistical differences (p<0.05) in weights, histological endpoints, and mRNA qRT-PCR array data.

## Results

### Preliminary Experiment

**Figure 2:**
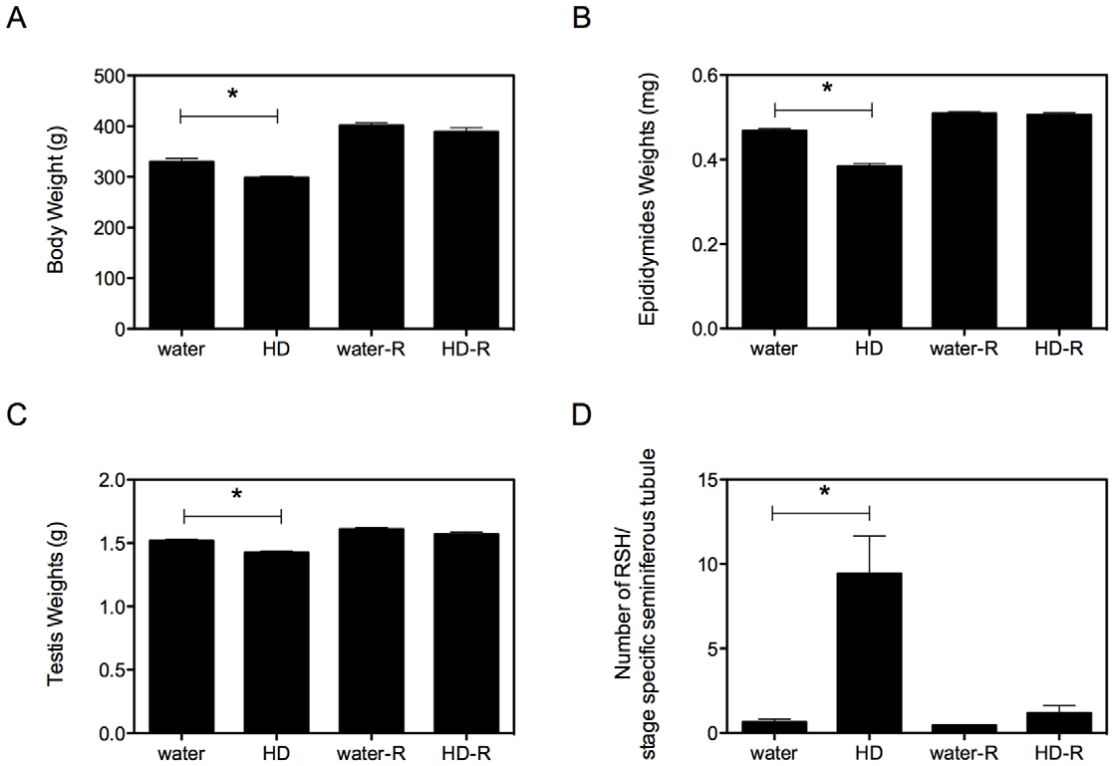
Preliminary experiment: weights and retained spermatid heads (RSH). In the Preliminary Experiment, body (A); epididymis (B); and testis (C) weights were recorded at necropsy for the four treatment groups (water and HD (black bars) and water-recovery and HD-recovery (water-R and HD-R; white bars). In addition, histological analyses of the testis were performed and the average number of RSH per stage-specific seminiferous tubule was determined (D). Data is presented as mean ± SE, and significance (* = p<0.001) was calculated for each pair of treatment and controls using Student’s unpaired two-tailed t-test. The RSH data was log-transformed prior to statistical analysis.

Rats were exposed to approximately 234 mg/kg/d HD during this study, similar to the previously reported range for low dose HD exposure [7]. This low HD dose induced a significant decrease in average body, testis, and epididymis weights after 3 months of exposure; and all of these effects resolved after 3 months of post-exposure recovery (Figure 2A–C). While various histopathological endpoints were assessed to determine the severity of testicular injury, the only effect observed after 3 months of exposure was a 14.5-fold increase in stage-specific spermatid head retention, which also resolved after recovery (Figure 2D).

The Affymetrix array platform provided whole-transcript coverage of 27,342 genes by ∼26 probes spread across the length of each gene. Utilizing the probe cell intensity values for the entire array, LIMMA analysis identified 128 transcripts with significantly altered levels in sperm after HD exposure and 112 of these had well-characterized gene annotations (Table S2). Of these 112 transcripts, 106 were elevated in the treated sperm, with fold change values as high as 2.64. No transcripts were significantly altered after 3 months of post-exposure recovery.

Of the list of 112 well-annotated significantly altered transcripts, 47 transcripts met the significance and fold change cutoffs (q<0.05 and |fold change|>1.5) for subsequent Ingenuity Pathway Analysis (IPA). After adjusting for multiple comparisons, a list of 17 significant high-level functional categories was generated that included cell death, cell cycle, cellular assembly and organization, and cellular growth and proliferation (Table 1).

**Table 1:** Preliminary Experiment: Functional Analysis of Microarray Data.

Category	Number of qRT-PCR Candidates with This Function
Cell Death	12
Cellular Assembly and Organization	9
Cellular Movement	9
Cell-To-Cell Signaling and Interaction	7
Cellular Development	7
Cellular Growth and Proliferation	7
Small Molecule Biochemistry	7
Cellular Compromise	6
Cellular Function and Maintenance	5
Cell Cycle	4
Lipid Metabolism	4
Molecular Transport	4
Carbohydrate Metabolism	3
Antigen Presentation	2
Post-Translational Modification	2
Free Radical Scavenging	1
Gene Expression	1

Note: IPA found that these functions were enriched in the microarray study (p<0.05 after adjusting for multiple comparisons). Candidates were selected from the microarray data based on the magnitude of their fold change and biological significance.

Twenty-nine differentially present mRNA transcripts were selected from the microarray data for further qRT-PCR analysis based on the strength of association, magnitude of change and biological function (Table S1).

### Time Course Experiment

Consistent with the Preliminary Experiment, the estimated daily dose of HD was approximately 224 mg/kg/d. As expected with the study design, body and epididymis weights increased as the animals grew over the time course experiment when compared to time point 0 (data not shown). There were no changes in testicular weights, HRSH, or serum inhibin B levels at any time point when compared to time point 0 (data not shown).

**Figure 3:**
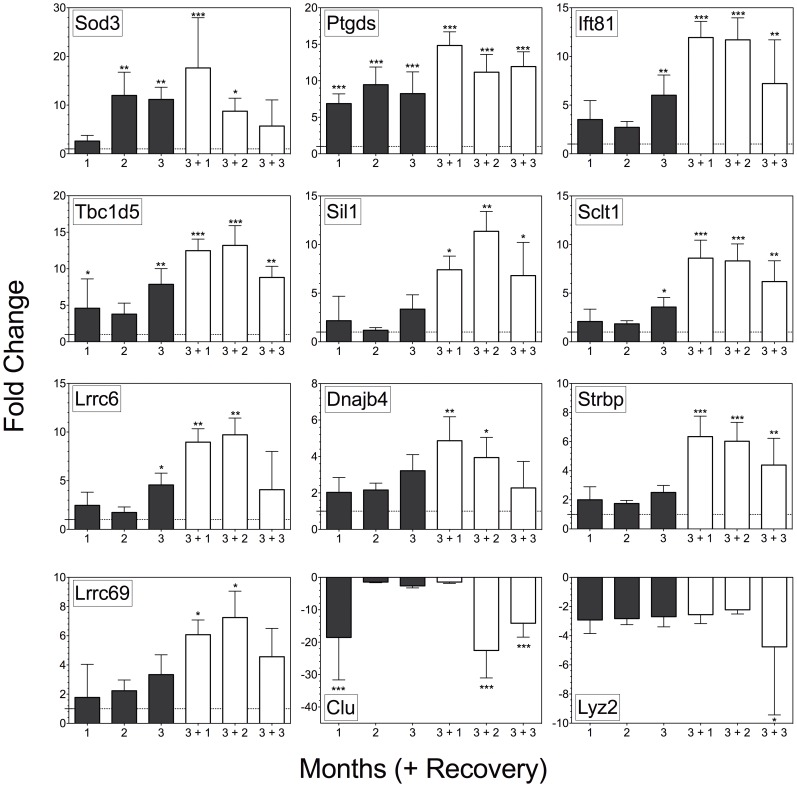
Time course experiment: transcript changes. Twelve of the 29 transcripts selected from the Preliminary Experiment showed significant alterations by qRT-PCR. ΔCT values were compared to time point 0 using one-way ANOVA with Dunnett’s correction for multiple comparisons (*** = p<0.001, ** = p<0.01, and * = p<0.05). Data are presented as mean ± range; the fold change range was generated using the formula 2^−ΔΔCT ± SE^. Black bars = treatment and white bars = recovery.

Analysis of the qRT-PCR-array data found that 12 of the 29 candidate transcripts were significantly altered at some time point when compared to time point 0, with fold changes ranging from −20 to 20 (Figure 3 and Table S3). Few transcripts (n = 3, 2, and 6) were altered after one, two, or three months of exposure, respectively. However, there was a robust sperm mRNA response in the post-exposure phase (n = 10, 11, and 8) at time points 3+1, 3+2, and 3+3, respectively (Figure 3 and Table S3). *Clu* and *Lyz2* were decreased during exposure, while the other 10 transcripts were increased.

**Figure 4:**
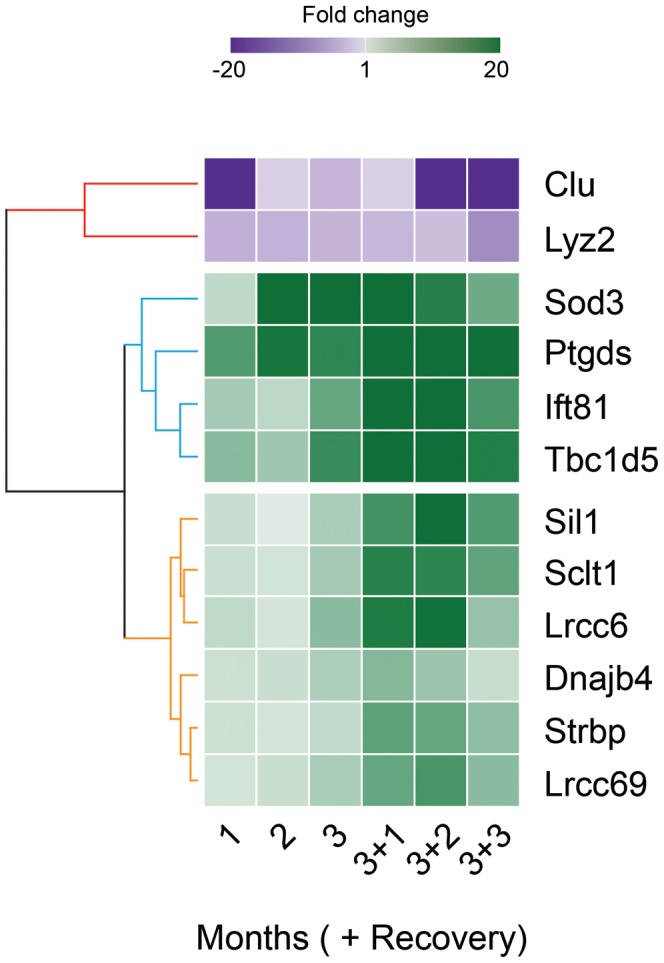
Time course experiment: heatmap displaying heirarchical clustering of qRT-PCR data. The fold changes determined in the qRT-PCR analysis (relative to time point 0) for the 12 transcripts altered at any of the six time points (columns) were clustered using Manhattan Distance. The 12 transcripts (rows) were separated via the direction of fold change (downregulated = red dendrogram; upregulated = blue and orange dendrograms). The upregulated transcripts segregated into two groups based upon the temporal onset of statistically different transcript levels, with early changing transcripts (blue dendrogram) separated from late changing transcripts (orange dendrogram). The intensity of the color for each transcript at each time point is related to the transcript level, as depicted by the bar at the top of the figure.

Heirarchical clustering of the 12 significantly altered transcripts from qRT-PCR-array data identified a major bifurcation in the transcript data (Figure 4), with transcripts separated by the direction of the fold change. The up-regulated cluster was further divided into two sub-clusters that were associated with the timing of the onset of the transcript change. Early changing transcripts were altered during the toxicant exposure (*Sod3*, *Ptgds*, *Tbc1d5*, and *Ift81*), and late changing transcripts were altered in sperm during post-exposure recovery (*Lrrc6*, *Sclt1*, *Sil1*, *Strbp*, *Lrrc69*, and *Dnajb4*).

Comparison of the qRT-PCR data from the Time Course Experiment with the Affymetrix array data from the Preliminary Experiment after 3 months of HD-exposure found that both platforms exhibited similar responses for the majority of transcripts. However, the qRT-PCR array had a greater dynamic range (−2.74 to 11.16 fold compared to −2.11 to 2.37 fold; Table S4). Only 6 of the 29 qRT-PCR array transcripts were significantly altered at 3 months when compared to time point 0 via one-way ANOVA with Dunnett’s correction for multiple comparisons (Table S4). Analyzing the qRT-PCR array data using Student’s unpaired two-tailed t-test comparing 0 and 3 months resulted in 10 significantly altered transcripts, with 4 additional transcripts approaching significance (p<0.09) (Table S4).

### Test Experiment

Body, testis, and epididymis weights of CBZ-exposed rats were similar to those of corn oil controls (data not shown). There were no changes in the number of HRSH in the testis after CBZ-exposure; however, CBZ increased the number of RSH in the testis 3.4-fold (p = 0.03). In addition, serum inhibin B levels were decreased in CBZ-exposed animals (p = 0.01). Eight of the 29 qRT-PCR array transcripts were altered in sperm after exposure to CBZ, with an additional two nearly significant (p<0.06). The fold changes for these transcripts ranged from −2.70 to 21.58 (Table 2). Of these 10 transcripts, 4 corresponded with the transcripts that were altered in the Time Course Experiment after HD exposure.

**Table 2:** Test Experiment: Fold Change Ratios for Altered Transcripts.

Transcript	Mean (range)^a^	p-values^b^
*Clu*	21.58 (26.94, 17.30)	<0.0001
*Sil1*	0.37 (0.51, 0.27)	0.0032
*Fank1*	2.44 (3.50, 1.70)	0.014
*Abi2*	2.50 (2.92, 2.15)	0.016
*Bag1*	2.69 (3.96, 1.83)	0.020
*Mfap3l*	2.29 (3.35, 1.56)	0.026
*Ift81*	0.47 (0.65, 0.33)	0.039
*Ptgds*	0.46 (0.63, 0.34)	0.047
*Bcl2l14*	2.26 (3.51, 1.46)	0.058
*Pim1*	1.95 (2.71, 1.40)	0.058

a The fold change range was generated using the formula 2^−ΔΔCT ± SE^.

b P-values were generated using Student’s unpaired two-tailed t-test.

## Discussion

This study demonstrates the utility of toxicogenomic approaches to detect evidence of testicular injury in sperm. The transcript changes initially identified by microarray analysis were confirmed using qRT-PCR arrays in two additional toxicant exposure paradigms. A time course experiment was performed to validate the alterations in sperm transcripts and to examine the time dependence of the HD effect. Over the time course of HD exposure and post-exposure recovery, we observed dynamic changes in sperm transcript content. Significant differences in steady state transcript levels for 12 of the 29 candidates were identified at various time points, with the most robust response occurring in the post-exposure recovery period. This was an unexpected finding because the microarray data suggested that the phenotypic and transcriptomic indicators of injury resolved 3 months after exposure cessation. The differences between the microarray and qRT-PCR recovery data are most likely due to differences in the sensitivity of the platforms, with the qRT-PCR arrays being more robust than the microarrays [31], [32] (Table S4).

Heirarchical clustering suggested a time-dependence to the transcript changes. The temporal dependence may result from effects to distinct germ cell populations during spermatogenesis, with later changing transcripts altered in spermatogonia, which have a long maturation sequence to undergo, and earlier changing transcripts disrupted in spermatids or spermatocytes, which take less time to mature into sperm. Sertoli cells support developing germ cells by creating a nurturing microenvironment that facilitates spermatogenesis. Sertoli cell toxicants alter this microenvironment and disrupt spermatogenesis. Our data suggests that exposed sperm contain abnormal transcript content. It is known that the pool of RNAs in mature spermatozoa represent a significant proportion of the RNAs synthesized prior to transcriptional arrest [33], so it is possible that these transcripts are leftover cell survival signals during early spermatogenesis. It is also possible that the regulation of these transcripts was purposefully altered to prepare sperm for embryogenesis in a stressful environment. On the other hand, the Sertoli cells or epididymal epithelial cells may be transferring transcripts to the sperm directly, and toxicant exposure could affect these processes. It has been previously hypothesized that sperm can take up foreign mRNAs, and *Clu*, a putative Sertoli cell mRNA, may be one of these transcripts [34]. Future work will investigate whether the epididymal epithelial cells or Sertoli cells directly provide the developing sperm with transcripts, or if the germ cells alter their own transcript content during the early stages of spermatogenesis in response to environmental changes.

The panel of sperm mRNA indicators identified by HD exposure was tested with another Sertoli cell toxicant, CBZ. No changes in body or reproductive weights were observed after low dose sub-chronic exposure to CBZ, but histology (an increase in RSH) and serum analysis (a decrease in inhibin B) provided evidence of subtle testicular injury. The HD-generated qRT-PCR array transcript panel was predictive of testicular injury resulting from CBZ exposure, with 10 of the 29 transcripts altered.

**Figure 5:**
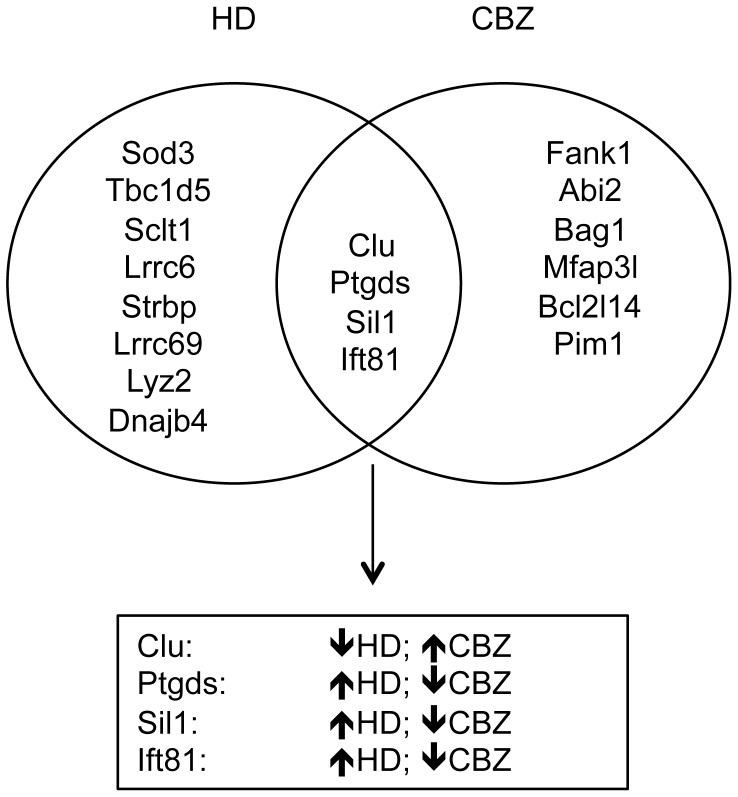
Venn diagram relating transcript profiles for HD and CBZ. A comparison of the sperm transcripts altered by HD and CBZ exposure identified 4 transcripts significantly altered by both exposures; however, the direction of change differed. The direction of the arrowhead indicates the direction of change of the transcript induced by toxicant exposure with up being upregulated and down being downregulated.

Although HD and CBZ both target the same cell-type within the testis (the Sertoli cell), the qRT-PCR data identified toxicant-specific alterations in the sperm transcriptome (Figure 5). This may be explained by the opposing actions of these two toxicants on the Sertoli cell microtubules, and highlights the potential of the qRT-PCR array panel to detect specific transcriptional signatures for different toxicants. HD promotes and stabilizes microtubule assembly by cross-linking tubulin, while CBZ inhibits microtubule assembly by binding the β-tubulin subunit of the αβ-tubulin heterodimer [9], [19]–[22]. Even though the toxicant mechanisms of actions differ, they ultimately produce similar phenotypic alterations in the seminiferous epithelium, including sloughing and RSH [9]. In this study, RSH were increased 14.5-fold by HD and 3.4-fold by CBZ. Our results suggest that RSH is an appropriate phenotypic anchor for determining Sertoli cell toxicity due to low dose sub-chronic exposures. However, the comparison of the RSH for the two toxicants highlights the fact that the doses we selected for each toxicant were not equipotent. The differences in the severity of the injury may also explain why the two toxicants produced dissimilar transcript profiles. Additional low-dose HD experiments are now underway in our laboratory to test this hypothesis.

Exposure to low dose HD induced alterations in sperm transcripts associated with stress response and apoptosis inhibition, possibly reflecting adaptive mechanisms invoked to maintain homeostasis in the developing germ cells. For example, SOD3 is an extracellular superoxide dismutase responsible for the removal of reactive oxygen species, and recent studies have suggested that SOD3 has an important role in regulating cellular signaling networks that reduce the development of injury and apoptosis [35]. In addition, DNAJB4 is a member of the DNAJ/HSP40 protein family, which participate in many cellular processes, including protein translation, folding, unfolding, translocation, and degradation; these proteins stimulate ATPase activation of heat shock 70 chaperone proteins to protect cells from stress [36], [37]. This hypothesis is consistent with our histopathology results that showed no changes in GC apoptosis after the 3-month exposure to HD.

Low dose CBZ exposure altered sperm transcripts associated with cell junctions and apoptosis. At higher doses, CBZ induces germ cell apoptosis in the testis and increases sloughing, which is the premature release of the germ cell from the Sertoli cell crypts. ABI2 is important for dynamic actin cytoskeleton remodeling at adherens junctions, which are important for Sertoli cell-germ cell interactions, including the movement of germ cells from the basement membrane to the lumen of the seminiferous tubules during spermatogenesis [38], [39]. BAG1 plays many roles in promoting cell survival and can interact with proteosomes and heat shock proteins to prevent cell death [40]. Our data showed an induction of these two transcripts, suggesting these mRNAs may reflect a germ cell response to prevent injury and apoptosis.

The qRT-PCR studies identified 4 transcripts (*Clu*, *Ptgds*, *Sil1*, and *Ift81*) that were found in sperm from both HD and CBZ exposed rats associated with functions important for stress response and spermatogenesis. CLU is a glycoprotein involved in many biological processes, including protecting cells from injury, mediating apoptosis, and influencing the differentiation and maturation of germ cells [41]. *Clu* mRNA has previously been detected by qRT-PCR in porcine spermatozoa, and sperm are hypothesized to deliver the *Clu* mRNA transcript to the oocyte to support its subsequent development after fertilization [42], [43]. PTGDS catalyzes conversion of prostaglandin H2 to prostaglandin D2, a major prostaglandin that regulates many bodily functions including sleep, body temperature, hormone release, and odor responses [44]. *Ptgds* mRNA has been measured in Sertoli cells and germ cells, and in the epididymis [44], where the protein product is an important component in the seminal fluid and may have a role in fertilization [45], [46]. SIL1 is an adenine nucleotide exchange factor for the heat-shock protein 70 member HSPA5/BiP [47], [48]. Defects in this gene have been implicated in Marinesco–Sjögren syndrome, which commonly presents with hypogonadism [48]. Heat-shock proteins are important for proper gametogenesis and embryogenesis, and induction of heat-shock protein-related mRNAs in sperm due to environmental stress could benefit the embryo after fertilization [49]. IFT81, also known as CDV-1, is necessary for the assembly and maintenance of eukaryotic cilia and flagella [50]. *Cdv-1R* mRNA is predominantly expressed in the testis with expression increasing with male sex maturation and onset of spermatogenesis [51]. In addition, *Cdv-1R* mRNA has been localized to the epididymis and may play an important role in sperm maturation [52]. Interestingly, the direction of change for all 4 transcripts differed between the two toxicants, and this may be due to the opposing actions of HD and CBZ on the microtubules within the Sertoli cell. Future research in our laboratory will further characterize these mRNA transcripts using comprehensive dose response studies and additional testicular toxicants.

Eighteen of the 29 sperm transcripts identified by microarrays as indicators of testicular toxicity were verified by subsequent qRT-PCR array analysis. We were unable to observe statistically significant changes for the other 11 sperm transcripts identified by microarray, and this may be due to the smaller sample size for the qRT-PCR studies (n = 4–9 compared to the ∼18–20). The 11 transcripts that did not change on the qRT-PCR array could also have been false positives on the microarray.

We have used a novel molecular based approach to identify sperm transcript changes after exposure to well characterized testicular toxicants. Given the novelty of this observation, we do not yet know whether these transcript alterations are shared manifestations of testicular injury in general across multiple species, or are chemical-specific in the rat. This study provides a proof-of-principle for the development of sperm indicators of testicular injury and suggests that measuring sperm mRNAs is a promising approach for screening toxicant-induced testicular injury. These data build upon our existing knowledge of testicular toxicity in animal models and develops the foundation required to extrapolate these observations to additional testicular toxicants and across species.

## Supporting Information

Table S1 Candidate Transcripts Selected from Microarray for qRT-PCR
(DOCX)

Table S2 Preliminary Experiment: Transcripts Altered in Sperm after 2,5-Hexanedione Exposure via Microarray Analysis
(DOCX)

Table S3 Time Course Experiment: Fold Change Ratios for Transcripts Significantly Changed after 2,5-Hexanedione Exposure
(DOCX)

Table S4 Time Course Experiment: Fold Change Comparisons After 3 Months of 2,5-Hexanedione Exposure
(DOC)
