# Supplementary material for: Sperm mRNA Transcripts Are Indicators of Sub-Chronic Low Dose Testicular Injury in the Fischer 344 Rat
Source: PLoS One. 2012 Aug 31;7(8):e44280. doi: 10.1371/journal.pone.0044280 (PMC3432073; doi:10.1371/journal.pone.0044280)
Supplement: Table S1 — Candidate Transcripts Selected from Microarray for qRT-PCR (DOCX) [file pone.0044280.s001.docx]

| **Table S1. Candidate Transcripts Selected from Microarray for qRT-PCR** | | |
| --- | --- | --- |
| **Transcript** | **Refseq #** | **Gene Ontology** |
| *Sil1* | NM_199376 | binding; unfolded protein binding |
| *Mtm1* | NM_001013047 | hydrolase activity; intermediate filament binding; phosphatase activity |
| *Lrrc6* | NM_001025659 | unknown |
| *Styxl1* | NM_001037788 | hydrolase activity; protein tyrosine/serine/threonine phosphatase activity; intracellular signal transduction |
| *Dcn* | NM_024129 | collagen binding; extracellular matrix binding; glycosaminoglycan binding; protein N-terminus binding |
| *Gas2* | XM_574454 | apoptotic process; cell cycle; cell cycle arrest; cellular component |
| *Tcp10b* | XM_217868 | unknown |
| *Mfap3l* | NM_001012049 | integral to membrane; plasma membrne |
| *Bcl2l14* | NM_001024338 | protein kinase binding; regulation of apoptosis |
| *Pim1* | NM_017034 | cell cycle; cell proliferation |
| *Lrrc69* | XM_001055887 | unknown |
| *Lyz2* | NM_012771 | hydrolase activity; lysozyme activity |
| *Phospho1* | XM_220877 | phosphatase activity |
| *Tax1bp1* | NM_001004199 | kinase binding; anti-apoptosis |
| *Dennd1a* | XM_231184 | SH3 domain binding; synaptic vesicle endocytosis |
| *Dnajb4* | NM_001013076 | heat shock protein binding; protein folding |
| *Clu* | NM_053021 | misfolded protein binding; anti-apoptosis; cellular response to transcription factor stimulus |
| *Bag1* | XM_216377 | chaperone binding; anti-apoptosis |
| *Ptgds* | NM_013015 | fatty acid biosynthesis; prostaglandin biosynthesis |
| *Bfar* | NM_001013125 | metal ion binding; anti-apoptosis |
| *Tpi1* | NM_022922 | isomerase activity; carbohydrate metabolic process |
| *Fank1* | NM_001008347 | unknown |
| *Sod3* | NM_012880 | metal ion binding; superoxide dismutase activity |
| *Vim* | NM_031140 | intermediate filament organization |
| *Ift81* | NM_199120 | cell differentiation; spermatogenesis |
| *Strbp* | NM_053416 | RNA binding; cell differentiation; cell component movement; spermatogenesis |
| *Tbc1d5* | XM_576503 | Rab GTPase activator activity |
| *Sclt1* | NM_153740 | clathrin binding; sodium channel regulator activity |
| *Abi2* | NM_173143 | SH3 domain binding; cellular component movement; cell migration |
