# Supplementary material for: Sperm mRNA Transcripts Are Indicators of Sub-Chronic Low Dose Testicular Injury in the Fischer 344 Rat
Source: PLoS One. 2012 Aug 31;7(8):e44280. doi: 10.1371/journal.pone.0044280 (PMC3432073; doi:10.1371/journal.pone.0044280)
Supplement: Table S2 — Preliminary Experiment: Transcripts Altered in Sperm after 2,5-Hexanedione Exposure via Microarray Analysis (DOCX) [file pone.0044280.s002.docx]

| **Table S2. Preliminary Experiment: Transcripts Altered in Sperm after 2,5-Hexanedione Exposure via Microarray Analysis** | | | |
| --- | --- | --- | --- |
| **Transcript** | **p-value** | **q-value** | **Fold Change** |
| *RGD1304694* | 0.000000085 | 0.0012 | 2.64 |
| *Mtm1* | 0.00000052 | 0.0025 | 2.37 |
| *Sil1* | 0.0000016 | 0.0040 | 2.29 |
| *LOC685184* | 0.000002 | 0.0045 | 2.13 |
| *Dcn* | 0.000013 | 0.011 | 2.09 |
| *Styxl1* | 0.000019 | 0.014 | 2.07 |
| *Gas2* | 0.0000044 | 0.0059 | 2.02 |
| *Lrrc6* | 0.0000094 | 0.0095 | 1.97 |
| *Mfap3l* | 0.000023 | 0.015 | 1.95 |
| *Tcp10b* | 0.000004 | 0.0059 | 1.94 |
| *Lyz2* | 0.00013 | 0.036 | 1.93 |
| *Bcl2l14* | 0.000024 | 0.015 | 1.91 |
| *Pim1* | 0.000031 | 0.018 | 1.90 |
| *Lrrc69* | 0.000052 | 0.025 | 1.89 |
| *Dennd1a* | 0.00007 | 0.028 | 1.87 |
| *Phospho1* | 0.000019 | 0.014 | 1.85 |
| *RGD1560258* | 0.0000044 | 0.0059 | 1.85 |
| *RGD1566314* | 0.0000017 | 0.0041 | 1.84 |
| *Tax1bp1* | 0.00008 | 0.030 | 1.81 |
| *Bfar* | 0.000091 | 0.030 | 1.80 |
| *RGD1563680* | 0.000068 | 0.027 | 1.80 |
| *Zfp407* | 0.000028 | 0.016 | 1.80 |
| *Dnajb4* | 0.00014 | 0.038 | 1.78 |
| *RGD1564140* | 0.000078 | 0.029 | 1.77 |
| *Ccdc46* | 0.00013 | 0.038 | 1.74 |
| *RGD1308023* | 0.00015 | 0.040 | 1.73 |
| *Tpi1* | 0.000066 | 0.027 | 1.70 |
| *Tbc1d5* | 0.000076 | 0.029 | 1.69 |
| *Sclt1* | 0.000016 | 0.012 | 1.68 |
| *Ift81* | 0.000056 | 0.026 | 1.67 |
| *Pou2f1* | 0.000024 | 0.015 | 1.65 |
| *Slc10a7* | 0.00000077 | 0.0025 | 1.63 |
| *Erc1* | 0.0000044 | 0.0059 | 1.62 |
| *Ubn2* | 0.000092 | 0.030 | 1.62 |
| *S100z* | 0.00000034 | 0.0025 | 1.62 |
| *LOC688916* | 0.00022 | 0.049 | 1.61 |
| *Sppl3* | 0.00000017 | 0.0017 | 1.61 |
| *LOC313149* | 0.000018 | 0.014 | 1.61 |
| *Abi2* | 0.00011 | 0.035 | 1.60 |
| *Tmem192* | 0.000093 | 0.030 | 1.59 |
| *RGD1309931* | 0.000089 | 0.030 | 1.58 |
| *LOC498330* | 0.000064 | 0.027 | 1.57 |
| *LOC500959* | 0.000071 | 0.028 | 1.57 |
| *Tpi1* | 0.000025 | 0.016 | 1.57 |
| *Fcer1g* | 0.00014 | 0.038 | 1.57 |
| *Slc10a7* | 0.00019 | 0.047 | 1.55 |
| *LOC681849* | 0.0000090 | 0.0094 | 1.54 |
| *Plekhk1* | 0.00021 | 0.049 | 1.54 |
| *Kif6* | 0.00012 | 0.035 | 1.54 |
| *Ctxn1* | 0.000000069 | 0.0012 | 1.52 |
| *RGD1307526* | 0.000063 | 0.027 | 1.52 |
| *LOC500700* | 0.00020 | 0.048 | 1.50 |
| *LOC307974* | 0.0000035 | 0.0059 | 1.50 |
| *Dnajc15* | 0.00015 | 0.038 | 1.48 |
| *Elof1* | 0.00019 | 0.047 | 1.48 |
| *Cd4* | 0.00012 | 0.035 | 1.47 |
| *Ppp1r9a* | 0.000087 | 0.030 | 1.46 |
| *Wdr7* | 0.00000049 | 0.0025 | 1.45 |
| *P2ry5* | 0.000026 | 0.016 | 1.42 |
| *Armc4* | 0.000064 | 0.027 | 1.42 |
| *Gimap4* | 0.000039 | 0.021 | 1.39 |
| *LOC311026* | 0.0000072 | 0.0080 | 1.39 |
| *Acer1* | 0.0000026 | 0.005 | 1.39 |
| *RGD1308023* | 0.000089 | 0.030 | 1.39 |
| *RGD1561537* | 0.000041 | 0.022 | 1.37 |
| *Ccr2* | 0.000024 | 0.015 | 1.37 |
| *Clec7a* | 0.00021 | 0.048 | 1.37 |
| *RGD1305469* | 0.000023 | 0.015 | 1.36 |
| *Spats1* | 0.000082 | 0.030 | 1.36 |
| *RGD1564887* | 0.000058 | 0.026 | 1.35 |
| *RGD1308023* | 0.0000099 | 0.0096 | 1.35 |
| *Kcng1* | 0.000012 | 0.010 | 1.35 |
| *Eaf2* | 0.000063 | 0.027 | 1.34 |
| *Tmeff1* | 0.000099 | 0.032 | 1.33 |
| *Fmn1* | 0.00014 | 0.038 | 1.32 |
| *Alg9* | 0.000012 | 0.010 | 1.31 |
| *Nol4* | 0.000061 | 0.027 | 1.30 |
| *Armc2* | 0.000043 | 0.022 | 1.30 |
| *Efcab5* | 0.00012 | 0.035 | 1.30 |
| *Hsf2bp* | 0.00012 | 0.036 | 1.29 |
| *Ptar1* | 0.0000039 | 0.0059 | 1.29 |
| *Znf532* | 0.0000080 | 0.0086 | 1.28 |
| *Gpatc2* | 0.000092 | 0.030 | 1.28 |
| *Ptprk* | 0.000094 | 0.030 | 1.27 |
| *Spsb3* | 0.000031 | 0.018 | 1.27 |
| *Cx3cr1* | 0.0000044 | 0.0059 | 1.27 |
| *C3* | 0.0000050 | 0.0063 | 1.26 |
| *RGD1560273* | 0.00020 | 0.048 | 1.26 |
| *Cacng7* | 0.000011 | 0.010 | 1.25 |
| *Aebp2* | 0.00019 | 0.047 | 1.24 |
| *Eya1* | 0.00011 | 0.035 | 1.23 |
| *Pcca* | 0.00013 | 0.037 | 1.21 |
| *Rps6ka5* | 0.0000013 | 0.0037 | 1.21 |
| *Klf12* | 0.000043 | 0.022 | 1.21 |
| *Nars2* | 0.000014 | 0.011 | 1.21 |
| *Gbp5* | 0.00019 | 0.047 | 1.20 |
| *Nek4* | 0.00015 | 0.040 | 1.20 |
| *LOC687517* | 0.00021 | 0.049 | 1.20 |
| *Ercc5* | 0.00000075 | 0.0025 | 1.20 |
| *Rims2* | 0.000039 | 0.021 | 1.19 |
| *Snapc1* | 0.00010 | 0.033 | 1.18 |
| *RGD1564943* | 0.000082 | 0.030 | 1.17 |
| *Axl* | 0.000055 | 0.026 | 1.17 |
| *Gli2* | 0.00019 | 0.047 | 1.16 |
| *Tmem116* | 0.00012 | 0.035 | 1.15 |
| *Rhobtb2* | 0.000088 | 0.030 | 1.14 |
| *Stmn1* | 0.00015 | 0.038 | -1.17 |
| *Uqcrc2* | 0.00015 | 0.038 | -1.18 |
| *Hdgfl1* | 0.000050 | 0.024 | -1.25 |
| *Sh2d4a* | 0.000038 | 0.021 | -1.27 |
| *Alox15b* | 0.000021 | 0.015 | -1.41 |
| *Clu* | 0.000061 | 0.027 | -2.11 |
