# Supplementary material for: Sperm mRNA Transcripts Are Indicators of Sub-Chronic Low Dose Testicular Injury in the Fischer 344 Rat
Source: PLoS One. 2012 Aug 31;7(8):e44280. doi: 10.1371/journal.pone.0044280 (PMC3432073; doi:10.1371/journal.pone.0044280)
Supplement: Table S3 — Time Course Experiment: Fold Change Ratios for Transcripts Significantly Changed after 2,5-Hexanedione Exposure (DOCX) [file pone.0044280.s003.docx]

| **Table S3. Time Course Experiment: Fold Change Ratios for Transcripts Significantly Changed after 2,5-Hexanedione Exposure** | | | | | | |
| --- | --- | --- | --- | --- | --- | --- |
| **Transcript** | **1** | **2** | **3** | **3+1** | **3+2** | **3+3** |
| *Clu* | 0.05*^a^*  (0.09, 0.03) | 0.69  (0.79, 0.60) | 0.38  (0.47, 0.31) | 0.69  (0.88, 0.54) | 0.04*^a^*  (0.06, 0.03) | 0.07*^a^*  (0.09, 0.05) |
| *Ptdgs* | 6.88*^a^*  (8.21, 5.76) | 9.47*^a^*  (11.88, 7.55) | 8.24*^a^*  (11.22, 6.05) | 14.82*^a^*  (16.69, 13.16) | 11.17*^a^*  (13.61, 9.17) | 11.95*^a^*  (13.97, 10.21) |
| *Sod3* | 2.61  (3.78, 1.80) | 11.98*^b^*  (16.75, 8.57) | 11.16*^b^*  (13.67, 9.12) | 17.64*^a^*  (27.95, 11.14) | 8.75*^c^*  (11.41, 6.71) | 5.69  (11.07, 2.93) |
| *Ift81* | 3.52  (5.46, 2.27) | 2.71  (3.32, 2.22) | 6.02*^b^*  (8.09, 4.47) | 11.94*^a^*  (13.60, 10.49) | 11.70*^a^*  (13.96, 9.81) | 7.22*^b^*  (11.70, 4.45) |
| *Tbc1d5* | 4.59*^c^*  (8.61, 2.45) | 3.78  (5.29, 2.70) | 7.86*^b^*  (10.01, 6.17) | 12.48*^a^*  (14.06, 11.07) | 13.19*^a^*  (15.90, 10.95) | 8.81*^b^*  (10.34, 7.50) |
| *Sclt1* | 2.08  (3.36, 1.29) | 1.85  (2.17, 1.57) | 3.57*^c^*  (4.56, 2.97) | 8.60*^a^*  (10.45, 7.08) | 8.32*^a^*  (10.07, 6.87) | 6.20*^b^*  (8.34, 4.61) |
| *Lrrc6* | 2.46  (3.82, 1.59) | 1.74  (2.30, 1.31) | 4.57*^c^*  (5.78, 3.61) | 8.96*^b^*  (10.34, 7.76) | 9.71*^b^*  (11.43, 8.25) | 4.08  (8.01, 2.08) |
| *Sil1* | 2.17  (4.68, 1.00) | 1.18  (1.46, 0.96) | 3.35  (4.83, 2.33) | 7.41*^c^*  (8.81, 6.23) | 11.36 *^b^*  (13.41, 9.62) | 6.81*^c^*  (10.22, 4.54) |
| *Strbp* | 2.00  (2.90, 1.38) | 1.75  (1.96, 1.55) | 2.51  (2.99, 2.10) | 6.35*^a^*  (7.75, 5.20) | 6.03*^a^*  (7.33, 4.96) | 4.39*^b^*  (6.23, 3.10) |
| *Lrrc69* | 1.77  (4.05, 0.78) | 2.23  (2.97, 1.68) | 3.34  (4.70, 2.38) | 6.07*^c^*  (7.08, 5.21) | 7.25*^c^*  (9.06, 5.80) | 4.56  (6.50, 3.20) |
| *Lyz2* | 0.34  (0.45, 0.26) | 0.35  (0.41, 0.31) | 0.37  (0.47, 0.29) | 0.39  (0.48, 0.32) | 0.45  (0.51, 0.40) | 0.21*^c^*  (0.42, 0.11) |
| *Dnajb4* | 2.03  (2.85, 1.45) | 2.16  (2.54, 1.84) | 3.22  (4.10, 2.52) | 4.87*^b^*  (6.18, 3.83) | 3.94*^c^*  (5.05, 3.08) | 2.28  (3.73, 1.39) |

Note: Data is presented as mean (range) fold change ratios. The fold change ratio range was generated using the formula 2^-ΔΔCT ± SE^. ΔCT values were compared to time point 0 using by one-way ANOVA with Dunnett’s correction for multiple comparisons: *^a^*, p <0.001; *^b^*, p < 0.01; and *^c^*, p <0.05.
