# Supplementary material for: Sperm mRNA Transcripts Are Indicators of Sub-Chronic Low Dose Testicular Injury in the Fischer 344 Rat
Source: PLoS One. 2012 Aug 31;7(8):e44280. doi: 10.1371/journal.pone.0044280 (PMC3432073; doi:10.1371/journal.pone.0044280)
Supplement: Table S4 — Time Course Experiment: Fold Change Comparisons After 3 Months of 2,5-Hexanedione Exposure (DOC) [file pone.0044280.s004.doc]

| **Table S4. Time Course Experiment: Fold Change Comparisons After 3 Months of 2,5-Hexanedione Exposure** | | |
| --- | --- | --- |
| **Transcript** | **Array** | **qRT-PCR** |
| *Clu* | -2.11 | -2.63 *^a, c^* |
| *Sod3* | 1.56 | 11.16 *^d, c^* |
| *Abi2* | 1.60 | 1.07 |
| *Vim* | 1.64 | -1.53 |
| *Strbp* | 1.65 | 2.51 *^a, c^* |
| *Fank1* | 1.66 | 1.68 |
| *Ift81* | 1.67 | 6.02 *^d, c^* |
| *Sclt1* | 1.68 | 3.57 *^a, c^* |
| *Tbc1d5* | 1.69 | 7.86 *^d, c^* |
| *Tpi1* | 1.70 | 1.68 |
| *Bag1* | 1.73 | 1.88 |
| *Ptgds* | 1.73 | 8.24 *^d, c^* |
| *Dnajb4* | 1.78 | 3.22 *^a, c^* |
| *Bfar* | 1.80 | -1.64 |
| *Tax1bp1* | 1.81 | -1.02 |
| *Phospho1* | 1.85 | -1.12 |
| *Dennd1a* | 1.87 | -1.16 |
| *Lrrc69* | 1.89 | 3.34 *^c^* |
| *Pim1* | 1.90 | 1.31 |
| *Bcl2l14* | 1.91 | 1.72 |
| *Lyz2* | 1.93 | -2.70 *^b^* |
| *Tcp10b* | 1.94 | 2.12 *^b^* |
| *Mfap3l* | 1.95 | 1.78 |
| *Lrrc6* | 1.97 | 4.57 *^a, c^* |
| *Gas2* | 2.02 | -2.74 |
| *Styxl1* | 2.07 | 2.85 *^b^* |
| *Dcn* | 2.09 | -2.07 |
| *Sil1* | 2.29 | 3.35 *^a, b^* |
| *Mtm1* | 2.37 | -1.05 |

Note:

*^a^* , p < 0.05 at any time point when compared to 0 via one-way ANOVA and Dunnett’s correction for multiple comparisons

*^b^*, p < 0.09 using student’s unpaired two-tailed t-test comparing 0 and 3

*^c^* , p < 0.05 using student’s unpaired two-tailed t-test comparing 0 and 3

*^d^*, p <0.05 at 3 months when compared to 0 via one-way ANOVA and Dunnett’s correction for multiple comparisons
